# Using Dominance Analysis to Identify the Most Important Dimensions of Safety Culture for Predicting Patient Safety

**DOI:** 10.3390/ijerph18157746

**Published:** 2021-07-21

**Authors:** Seung Eun Lee, V. Susan Dahinten

**Affiliations:** 1 Mo-Im KIM Nursing Research Institute, College of Nursing, Yonsei University, Seoul 03722, Korea; 2 School of Nursing, University of British Columbia, Vancouver, BC V6T 2B5, Canada; Susan.Dahinten@ubc.ca

**Keywords:** patient safety culture, patient safety, nurses, dominance analysis

## Abstract

Studies have demonstrated associations between safety culture and patient safety based on the perceptions of healthcare professionals, but limited attention has been given to the perceptions of nurses. Moreover, most studies have used regression modeling, an approach that limits researchers’ ability to identify the most important predictors of patient safety due to intercorrelations among predictors in the model. Therefore, the purpose of this study was to examine the effects of seven dimensions of safety culture on nurse-rated patient safety and identify the relative importance of these dimensions for predicting patient safety. This correlational study used data from the Agency for Healthcare Research and Quality’s 2018 Hospital Survey on Patient Safety Culture. Data from 13,031 nurses working in surgical areas of 443 hospitals in the United States were examined using logistic regression and dominance analysis. Staffing adequacy was the strongest predictor of patient safety, followed by hospital management support for patient safety and organizational learning/continuous improvement. However, dominance analysis showed that hospital management support for patient safety was the most important predictor rather than staffing adequacy. Nurse managers and hospital administrators should role model a culture of safety and demonstrate their valuing of patient safety by providing sufficient resources, listening to and valuing staff suggestions regarding patient safety, and providing feedback about organizational changes to improve patient safety.

## 1. Introduction

Safety culture, one aspect of organizational culture, is widely recognized as a critical factor for improving and maintaining patient safety [1,2,3]. Safety culture has been defined as the shared values, beliefs, and norms that shape an organization’s priorities and drive safety-related behaviors to minimize patient harm [4]. Assessing the current status of safety culture in healthcare organizations, and identifying the dimensions of safety culture that are the most important predictors of patient safety, are the first steps to improving that culture and enhancing patient safety. Although always important, this is even more important during critical periods such as the COVID-19 pandemic when human and other resources are stretched further than usual.

Healthcare professionals’ perceptions of safety culture and their association with patient safety have been examined in hospitals around the world, for example, in Canada [5], the United States [6], the Netherlands [7], Jordan [8], Taiwan [9], China [10,11], and Saudi Arabia [12]. Although safety culture has been linked with patient safety outcomes, a recent literature review revealed that research findings regarding these relationships have been inconsistent [13]. Possible reasons for the inconsistencies include the aggregation of data across professions and clinical specialties, the infrequent use of a theoretical framework, and limited analytic approaches [13].

Studies have demonstrated associations between safety culture and patient safety based on the perceptions of healthcare professionals, but limited attention has been given to the perceptions of nurses. Nurses play an especially important role in patient safety due to their 24 h responsibilities for patient care, patient surveillance, and care management. Thus, their perceptions of safety culture and patient safety may provide a more comprehensive or accurate safety indicator in healthcare organizations [6,12]. Moreover, previous research has found that nurses’ perceptions of safety culture differ from those of other healthcare professionals such as physicians [7,9,11], as well as laboratory, clinical, and non-medical workers [14]. Therefore, nurses’ perceptions of safety culture should be examined separately from those of others, but this is often not the case (e.g., [15]). Furthermore, even when perceptions of safety culture have been measured separately and found to vary by healthcare profession, the data are often aggregated in the correlation and regression analyses (e.g., [10,16]). An exception is a study by Verbeek-Van Noord et al. [7] that identified differences in the correlations between dimensions of safety culture and patient safety when analyzed separately for physicians and nurses. In addition, previous studies have included samples drawn from various work specialties [4,17] despite evidence that there are greater differences in perceptions of safety culture between work areas than between hospitals [18,19]. Thus, it is important to examine associations between safety culture and patient safety among nurses working in similar specialties, an approach not emphasized in previous studies.

Safety culture is a multidimensional concept; therefore, it is important to identify the specific aspects of safety culture that are significantly associated with patient safety [20]. Most previous research on associations between nurses’ perceptions of safety culture and patient safety have used regression modeling (e.g., [7,8,21]), an approach that limits researchers’ ability to identify the most important predictors of patient safety due to intercorrelations among the various predictors in the model [22]. This, in turn, limits healthcare decision-makers’ ability to identify and focus their interventions on the most influential factors for improving patient safety. To overcome this limitation, an analytical method such as dominance analysis, which identifies predictors’ relative importance in a statistical model, can be used to supplement regression analysis [23]. Dominance analysis accounts for a variable’s total, direct, and partial effects in terms of its contribution to overall variance; thus, this analytic method can accurately identify the contributions of individual predictors that cannot be determined by multiple regression alone [22,24]. Therefore, to identify the predictors of greatest importance to nurse-rated patient safety, this study employed dominance analysis in addition to regression modeling.

Research on safety culture and patient safety outcomes has been critiqued as atheoretical [13]. This study was guided by Donabedian’s structure–process–outcome (SPO) model of quality of care [25]. Structure refers to characteristics of the healthcare setting such as organizational structure or nurse–patient ratios, process refers to activities performed in providing and receiving care, and outcome refers to the effects of care. Based on the SPO model and previous research, this study hypothesized that various structural aspects of safety culture (e.g., the adequacy of staffing resources, hospital leaders’ valuing of safety culture, and mechanisms for continuous organizational learning) could impact nurse-rated patient safety (outcome) in hospitals. Therefore, the purpose of this study was to (1) examine relationships between seven structural aspects of safety culture and nurse-rated patient safety and (2) identify the relative importance of these aspects for predicting patient safety by applying dominance analysis.

## 2. Materials and Methods

### 2.1. Design and Sample

This cross-sectional, correlational study used publicly available, de-identified data from the 2018 Agency for Healthcare Research and Quality Hospital Survey on Patient Safety Culture (HSOPSC). The dataset includes survey data voluntarily submitted by hospitals in 50 states and territories of the United States (i.e., a form of convenience sampling). To achieve the aims of the current study, only data from registered nurses (RN) who were providing direct patient care in one specialty—surgical care—were included in the analysis. Thus, the sample included 13,031 RNs from 443 hospitals. Ethical approval was obtained from the institutional review board of the first author’s institution.

### 2.2. Measures

The HSOPSC collects data regarding hospital staff perceptions of patient safety issues as well as limited demographic information [26]. Most survey items employed 5-point response scales measuring respondent agreement (‘strongly disagree’ to ‘strongly agree’) or frequency (‘never’ to ‘always’). The overall HSOPSC and its dimensions have been found to be reliable and valid measures at the individual, unit, and hospital levels [27]. Additionally, the instrument has been shown to be reliable in samples of healthcare workers in central [28], northeast [18], and various regions [29] of US healthcare settings.

**Outcome variable.***Nurse-rated patient safety* was measured using a single item asking nurses to assign an overall grade to patient safety in their work units. This item was measured on a 5-point response scale ranging from ‘failing’ to ‘excellent.’ In the current study, this variable was re-categorized as a dichotomous variable as 0 (failing, poor, or acceptable) and 1 (very good or excellent). This item has been used as an outcome variable in previous research [11,27,30] and found to be a reliable measure of patient safety [7].

**Predictor variables.** Although the HSOPSC measures 12 dimensions of safety culture, in this study, we used only seven HSOPSC subscales—those that measure structural characteristics of hospitals. Other subscales in the HSOPSC indicate actions by frontline staff (e.g., teamwork and error reporting) and are more consistent with ‘process’ variables in Donabedian’s SPO model [25]. Each of the seven predictor variables in our model represented one dimension of safety culture as follows. *Supervisor/manager expectations and actions promoting safety* (4 items) assessed nurses’ perceptions of their managers’ expectations and actions for improving patient safety (e.g., ‘My supervisor/manager says a good word when he/she sees a job done according to established patient safety procedures’). *Hospital management support for patient safety* (3 items) assessed nurses’ perceptions of their hospital management’s support for improving patient safety (e.g., ‘Hospital management provides a work climate that promotes patient safety’). *Staffing adequacy* (4 items) assessed nurses’ perceptions of having enough staff to provide the best patient care. *Communication openness* (3 items) assessed nurses’ perceptions of feeling safe to express their views and pose questions about patient safety to persons with more authority. *Nonpunitive response to error* (3 items) assessed nurses’ perceptions of being punished when they committed an error and reported it (e.g., ‘When an event is reported, it feels like the person is being written up, not the problem’). The term *error* refers to ‘any act of commission (doing something wrong) or omission (failing to do the right thing) that exposes patients to a potentially hazardous situation’ [31] (pp.2). The error may or may not result in patient harm. *Feedback and communication about error* (3 items) assessed nurses’ perceptions of receiving feedback on their event reports and having discussions about ways to prevent recurrence of errors. *Organizational learning*—*continuous improvement* (3 items) assessed nurse-perceived organizational commitment to continuous improvement in patient safety. A mean score was calculated for each dimension of safety culture, with higher scores indicating higher levels of the dimension. For the study sample, Cronbach’s alpha values for the predictor variables were above 0.70 (ranging from 0.76 to 0.83) for each subscale except staffing adequacy, which yielded an alpha value of 0.67.

**Control variables.** We controlled for two hospital-related variables in the data analysis. One was size (small: 99 or fewer beds; medium: 100 to 199 beds; large: 200 or more beds), and the second was teaching status (teaching versus non-teaching).

### 2.3. Data Analysis

Descriptive statistics were used to describe the demographic characteristics of the sample and key study variables. Pearson and Point-Biserial correlations were used to examine the direction and strength of bivariate relationships between variables. Logistic regression was employed to identify significant predictors of nurse-rated patient safety. Logistic regression with the cluster option was applied to account for the clustering of nurses within hospitals [32]. Finally, dominance analysis was used to determine the relative importance of each predictor to the outcome. We used the dominance analysis procedure developed by Azen and Traxel [33] for use with logistic regression. This procedure is an extension of the approach developed earlier [34,35] for determining predictor importance in linear regression. Dominance analysis compares pairs of predictors across all subsets of the predictors in a model to determine the additional contribution that each predictor makes to the prediction model. A predictor is considered dominant or more important when it makes a larger contribution to every possible subset of predictors than any of the other predictors. Respondents were excluded from analysis in cases where all items within a dimension were missing. All statistical procedures, including dominance analysis, were conducted using STATA version 13 (StataCorp, LP, College Station, TX, USA) with a statistical significance level of *p* < 0.05.

## 3. Results

### 3.1. Participant Characteristics and Descriptive Statistics on Key Variables

Table 1 presents the characteristics of the total sample of 13,031 RNs and 443 hospitals. Approximately 32% of the RN participants had worked as a RN for 5 years or less, whereas 38% had 16 years or more experience in nursing. Approximately 57% of the RNs (53%) worked less than 40 h per week. Most hospitals (65%) were non-teaching hospitals, and 43% had 200 or more beds. Table 2 shows the descriptive statistics for key variables in this study. Approximately one-quarter of RNs (27%) rated patient safety in their work units as less than very good or excellent. Bivariate correlation analyses showed that all safety culture dimensions were significantly and positively associated with nurse-rated patient safety, with correlation values ranging from 0.37 to 0.67.

### 3.2. Logistic Regression Results

Binary logistic regression was performed to examine the effects of the seven safety culture dimensions on nurse-rated patient safety. The effects were first analyzed separately and then jointly. As shown in Table 3, after controlling for hospital characteristics and the clustering of nurses within hospitals, staffing adequacy (odds ratio (OR) = 2.57, 95% confidence interval (CI) = 2.35–2.81) was the strongest predictor of patient safety, followed by hospital management support for patient safety (OR = 2.41, CI = 2.19–2.66), organizational learning—continuous improvement (OR = 2.04, CI = 1.81–2.30), communication openness (OR = 1.75, CI = 1.58–1.93), supervisor/manager expectations and actions promoting safety (OR = 1.45, CI = 1.30–1.62), and feedback and communication about error (OR = 1.29, CI = 1.16–1.44). RNs who perceived that they had sufficient staff to provide quality patient care were about two and one-half times more likely to report patient safety in their units as being very good or excellent than those who perceived inadequate staffing levels. Furthermore, RNs who perceived higher levels of support from hospital management for patient safety were more than two times more likely to report patient safety as being very good or excellent than those who perceived lower levels of management support. Similarly, RNs who perceived that their organizations made continuous efforts to improve patient safety were two times more likely to report patient safety as being very good or excellent than those who perceived lower levels of continuous organizational commitment. In addition, RNs were more likely to assign higher patient safety grades to their units when they perceived higher levels of communication openness, unit supervisor/manager expectations and actions for patient safety, and feedback and communication about errors. Nonpunitive response to error was found to be significantly associated with nurse-graded patient safety in an unadjusted model, but after controlling for other variables, the association became non-significant.

### 3.3. Dominance Analysis Results

As shown in Table 3, the rankings that resulted from dominance analysis varied slightly from that of the regression analysis in that hospital management support for patient safety was found to be the most influential predictor, rather than staffing adequacy. The rankings of communication openness and supervisor/manager expectations and actions promoting safety were also reversed. Dominance analysis of the seven safety culture dimensions revealed that hospital management support for patient safety, staffing adequacy, and organizational learning—continuous improvement were the three highest-ranked predictors for nurse-rated patient safety, accounting for 57.15% of the predicted variance (23.13%, 18.13%, and 15.89%, respectively). Nonpunitive response to error, a non-significant predictor in the adjusted logistic regression model, was the lowest-ranked predictor in the dominance analysis, accounting for 5.8% of the predicted variance.

## 4. Discussion

Nurses are likely to be the first to notice issues regarding patient safety in clinical settings, and thus their perspectives on patient safety in specific work units merit careful examination. Researchers have found that safety culture is an important organizational factor in improving patient safety, but the effects of specific dimensions of safety culture on nurse-assessed patient safety have received little attention. This study investigated associations between seven structural aspects of safety culture and nurse-rated patient safety in the surgical units of 443 hospitals and identified the relative importance of the seven predictors for predicting patient safety through the use of dominance analysis.

Logistic regression results indicated that all structural aspects of safety culture were uniquely predictive of patient safety except for nonpunitive response to error. Staffing adequacy, hospital management support for patient safety, and organizational learning-continuous improvement were found to be the three strongest predictors, each with odds ratios greater than 2.0. Dominance analysis also identified these three factors as the most important predictors of patient safety, but in a different order of dominance.

The dominance analysis results of this study underscored the importance of hospital management support for safety culture and continuous organizational improvements in patient safety. Similar to previous research, this study found that nurses were more likely to assign higher patient safety grades when they perceived that hospital management prioritized patient safety [7,8,36]; that their unit supervisors invited and seriously considered staff suggestions for enhancing patient safety [8,36]; and that their organizations strived to continuously learn from patient safety-related errors, make appropriate changes, and assess their effects [8,33]. In another study [3], participants reported that that they were motivated to improve patient safety culture after receiving feedback from hospital administration on safety culture. Together, these findings emphasize the importance of strong, competent, and visible leadership for creating and improving safety culture in healthcare organizations. Hospital management plays a key role in establishing the culture and commitment required to address systemic causes of patient safety issues [36]. Management practices influence staff perceptions and behaviors regarding safety matters, and thus management can direct cultural shifts toward greater safety [37].

The results of this study situated within the US hospital context are consistent with previous global research reporting a positive association between staffing adequacy and nurse-rated patient safety in Lebanese [36], Swedish [38], and Jordanian hospitals [8]. Thus, these findings highlight the importance of health policies that ensure adequate nurse staffing to support safe patient care [26,38].

As in previous research [7,8], the current study found that feedback and communication about errors, as well as communication openness, were positively associated with RNs’ views of patient safety in their units. RNs viewed patient safety as very good or excellent when they received feedback on event reports, discussed ways to prevent recurring errors or near misses, and felt free to share their views and questions about patient safety with hospital leaders. These findings show the importance of policies that create safe environments in which nurses can express their safety concerns and suggestions. Managers and unit supervisors should allocate the time and space resources needed to seek input from nursing staff and recognize their contributions to patient safety.

Despite an assumption that a nonpunitive response to error would be important to RNs’ safety perceptions, this aspect of safety culture did not make a unique contribution to the explanation of nurse-rated patient safety. By way of contrast, in a previous study, nonpunitive response to error was significantly and positively associated with patient safety assessments made by 6087 hospital employees from various professions and departments [36]. This inconsistency may be attributable to the current study sample being limited to direct-care RNs working in surgical units. In a study that examined the effects of various aspects of safety culture on staff-assessed patient safety in 33 emergency departments in the Netherlands, Verbeek-Van Noord et al. [7] found that physicians tended to rate patient safety significantly higher than nurses. This finding indicates that patient safety perceptions can differ among healthcare disciplines. As in the current study, a nonpunitive environment was not significantly associated with nurse-rated patient safety in Jordanian hospitals [8]. The inconsistent findings regarding nonpunitive response to error indicate a need for more research, particularly in regard to discipline-specific perceptions of patient safety.

The strengths of this study were its large sample drawn from hundreds of hospitals and focus on direct-care nurses working in a single clinical specialty. However, the cross-sectional survey data preclude causal inferences. A second limitation is that all data were based on nurses’ perceptions. Although data from objective measures of patient safety measures such as incident reports were not available, researchers have demonstrated that nurse-assessed patient safety is a reliable and valid indicator of safety in clinical settings [39]. Incident reports (also called event reports) include reports of errors and adverse events (i.e., patient harm that has arisen from medical care) [31]. Third, perceptions of safety culture and patient safety may vary with the healthcare context (e.g., specialties or regions), so the findings of this study should be generalized with caution. Finally, data were not available to control for unmeasured factors (e.g., nurses’ education level, safety culture improvement programs in hospitals, incident reporting systems) that could have contributed to the relationships found in this study.

Creating and improving safety culture is key to enhancing patient safety in healthcare organizations. Administrators and clinical leaders should collaborate with staff to create a shared vision of safety culture that can be reflected in the policies and guidelines of the organization. For example, one essential element of a system-wide safety culture is a policy that mandates open communication about patient safety issues among staff and managers and specifies how feedback is to be provided when a safety event is reported. Hospital administrators and unit managers should implement policies that promote a learning culture in which staff members are willing to share their experiences and errors so that all can learn from them. Such policies will create opportunities for healthcare organizations to strengthen safety-related systems by systematically analyzing the root causes of safety problems [40] and then taking corrective action. Additionally, policies and guidelines for providing routine patient safety training—not only for staff but also for leaders at all levels—are needed to maintain and enhance a culture of safety. As safety culture has been shown to vary between hospitals and departments [41], unit-specific and hospital-specific strategies for improving safety culture should be considered. For this, hospital leaders and unit managers should examine the current state of safety culture in their organizations to identify and prioritize areas needing improvement. Finally, efforts should be made at the national level. A nation-wide safety program, including evidence-based safety standards and nonpunitive error reporting systems, should be developed so that safety culture across the healthcare system can be enhanced [3] Even though nonpunitive error reporting was not found to be uniquely predictive over and above other safety culture dimensions in our study, it was associated with patient safety and may be one of the easier interventions to implement.

## 5. Conclusions

Patient safety remains one of the greatest challenges in health care globally [42]. Assessing the current status of safety culture in healthcare organizations, and identifying the dimensions of safety culture that are the most important predictors of patient safety, are the first steps to improving that culture and enhancing patient safety. Although always important, this is even more important during critical periods such as the COVID-19 pandemic when human and other resources are stretched further than usual.

This study provides empirical evidence that organizational safety culture is significantly associated with nurse-assessed patient safety. All structural aspects of safety culture, with the exception of nonpunitive response to error, were uniquely predictive of patient safety, but nurses were much more likely to rate patient safety higher in their surgical units when they perceived higher levels of management support for patient safety, staffing adequacy, and continuous organizational improvement in patient safety. This suggests that improvements to patients need to start with the top levels of hospital administration, notwithstanding the importance of unit-level influences.

## Figures and Tables

**Table 1:** Characteristics of nurses and hospitals.

	Characteristic	Category	*f* (%)
Nurses (*N* = 13,031)	Experience (years)	Less than 1	788 (6.05)
		1 to 5	3334 (25.59)
		6 to 10	2362 (18.13)
		11 to 15	1576 (12.09)
		16 to 20	1469 (11.27)
		21 or more	3502 (26.87)
	Work hours (per week)	Less than 20	464 (3.56)
		20 to 39	6947 (53.31)
		40 to 59	5081 (38.99)
		60 to 79	356 (2.73)
		80 to 99	140 (1.07)
		100 or more	3 (0.02)
		Missing	40 (0.31)
Hospitals (*N* = 443)	Teaching status	Non-teaching	289 (65.23)
		Teaching	154 (34.77)
	Number of beds	Less than 100	139 (31.38)
		100 to 199	114 (25.73)
		200 or more	190 (42.89)

**Table 2:** Descriptive statistics for key predictors and outcome variable (N = 13,031).

Predictors	*M* (SD)	*f* (%)
Supervisor/manager expectations and actions promoting safety	3.88 (0.86)	
Hospital management support for patient safety	3.59 (0.92)	
Organizational learning—continuous improvement	3.77 (0.73)	
Communication openness	3.72 (0.81)	
Feedback and communication about error	3.80 (0.83)	
Nonpunitive response to error	3.27 (0.94)	
Staffing adequacy	3.31 (0.83)	
Outcome variable		
Nurse-rated patient safety		
Very good or excellent		9306 (73.19)
Failing, poor, or acceptable		3409 (26.81)

*M* = mean; *SD* = standard deviation.

**Table 3:** Logistic regression and importance of predictors on nurse-rated patient safety.

	Unadjusted OR (95% CI)	Adjusted OR (95% CI) ^a^	Standardized Dominance Statistic ^b^	Ranking
Supervisor/manager expectations and actions promoting safety	4.90 (4.53–5.31) ***	1.45 (1.30–1.62) ***	0.1304	4
Hospital management support for patient safety	5.59 (5.10–6.13) ***	2.41 (2.19–2.66) ***	0.2313	1
Organizational learning—continuous improvement	7.68 (6.98–8.46) ***	2.04 (1.81–2.30) ***	0.1589	3
Communication openness	5.16 (4.77–5.89) ***	1.75 (1.58–1.93) ***	0.1255	5
Feedback and communication about error	4.47 (4.15–4.83) ***	1.29 (1.16–1.44) ***	0.1080	6
Nonpunitive response to error	2.74 (2.57–2.92) ***	0.99 (0.92–1.07)	0.0580	7
Staffing adequacy	4.64 (4.31–4.99) ***	2.57 (2.35–2.81) ***	0.1813	2

OR = odds ratio; CI = confidence interval. ^a^ Adjusted odds ratios from logistic regression models, controlling for hospital size, teaching status, and clustering of nurses within hospitals. ^b^ Standardized dominance statistics do not total to 1 due to rounding. *** *p* < 0.001.

## Data Availability

The SOPS^TM^ dataset used in this analysis may be obtained from the US Agency for Healthcare Research and Quality (AHRQ).
